# Comparative Effects of Two Forms of Chitosan on Selected Phytochemical Properties of *Plectranthus amboinicus* (Lour.)

**DOI:** 10.3390/molecules28010376

**Published:** 2023-01-02

**Authors:** Maria Stasińska-Jakubas, Barbara Hawrylak-Nowak, Magdalena Wójciak, Sławomir Dresler

**Affiliations:** 1 Department of Botany and Plant Physiology, Faculty of Environmental Biology, University of Life Sciences in Lublin, Akademicka 15, 20-950 Lublin, Poland; 2 Department of Analytical Chemistry, Medical University of Lublin, Chodźki 4a, 20-093 Lublin, Poland; 3 Department of Plant Physiology and Biophysics, Institute of Biological Science, Maria Curie-Skłodowska University, 20-033 Lublin, Poland

**Keywords:** chitosan, Indian borage, phenolic compounds, biotic elicitor, secondary metabolites

## Abstract

In response to stress factors, plants produce a wide range of biologically active substances, from a group of secondary metabolites, which are applied in medicine and health prophylaxis. Chitosan is a well-known elicitor affecting secondary metabolism in plants, but its effect on the phytochemical profile of *Plectranthus amboinicus* has not been assessed yet. In the present experiment, the effectiveness of the foliar application of two forms of chitosan (chitosan suspension or chitosan lactate) was compared in order to evaluate their potential to induce the accumulation of selected polyphenolic compounds in the aboveground parts of *P. amboinicus*. It was shown that chitosan lactate had substantially higher elicitation efficiency, as the use of this form exerted a beneficial effect on the analysed quality parameters of the raw material, especially the content of selected polyphenolic compounds (total content of polyphenols, flavonols, anthocyanins, and caffeic acid derivatives) and the free radical-scavenging activity of extracts from elicited plants. Concurrently, it had no phytotoxic effects. Hence, chitosan lactate-based elicitation can be an effective method for optimisation of the production of high-quality *P. amboinicus* raw material characterised by an increased concentration of health-promoting and antioxidant compounds.

## 1. Introduction

The current trends in the promotion of a healthy lifestyle and the simultaneous increase in the prevalence of lifestyle diseases have contributed to the growing interest of many industries in both high-quality herbal raw materials and the contents of health-enhancing substances in plants, especially those with antioxidant properties [1]. Those plant chemical compounds have the ability to protect the body against the harmful effects of oxidative stress and prevent the risk of different cardiovascular, metabolic, and degenerative diseases, cancers, or premature aging [2,3]. For this reason, it is important not only to search for solutions to stimulate the production of desired secondary metabolites but also to investigate the chemical composition and properties of plant species that have not been used on a larger scale so far [3].

One of the effective methods for enhancement of the phytoaccumulation of bioactive compounds and thus improvement of the quality of raw materials involves elicitation with various types of substances and factors used as the so-called elicitors of plant defence reactions [4]. As indicated in current literature reports, elicitors stimulate receptors on the surface of the cytoplasmic membrane and activate a number of plant defence mechanisms, leading to the stimulation of secondary metabolism pathways and production of compounds that contribute to plant survival in stress conditions [5,6]. An increasingly frequently used biotic elicitor is chitosan, which can potentially be applied for the optimisation of plant production due to its natural origin, favourable physicochemical properties, and high biological activity, with the most important antioxidant and antimicrobial effects, as well as the regulation of plant growth, development, and resistance. Chitosan is a polysaccharide biopolymer derived from the partial deacetylation of chitin, obtained most often from marine-processing waste and from insect exoskeletons and fungal cell walls [7,8,9]. Additionally, an important issue in the context of elicitation is that the use of chitosan helps to improve the yield of many phytochemical antioxidant compounds, e.g., substances from the group of polyphenols, terpenoids, naphthoquinones, and alkaloids [7,10].

Polyphenolic compounds constitute a large group of secondary metabolites comprising over 8000 substances, with phenolic acids, flavonoids, isoflavonoids, stilbenes, and lignans as the most important compounds. In plants, these substances perform important protective functions against unfavourable biotic and abiotic environmental factors [11,12,13]. However, most polyphenols also exert a beneficial effect on the human organism through their antioxidant, anti-inflammatory, antiviral, anti-proliferative, hepatoprotective, nephroprotective, antidepressant, immunomodulatory, and anticancer activity. They can be effective e.g., in the treatment of cardiovascular diseases, skin diseases, diabetes, cancer, and neurodegenerative diseases, especially for the prophylaxis and treatment of Alzheimer’s disease [11,14,15,16,17]. Additionally, polyphenols can modulate energy metabolism, thereby exerting a positive effect on general well-being, delaying the aging process, and reducing the risk of age-related diseases [18]. These compounds play a significant role not only for plants that produce them, but also for people who use their health-beneficial properties. The importance of polyphenols and their connection with plant stress responses and secondary resistance in animals is often emphasised in the literature as xenohormesis hypothesis. It assumes that some plant chemical compounds are able to allow heterotrophs to adapt to changing environmental conditions by inducing biological responses. Therefore, xenohormesis is in fact the final pharmacological effect initiated by plant adaptation [19,20,21]. Given the wide spectrum of activity and the high abundance of this group of compounds in the chemical composition of *Plectranthus amboinicus* (Lour.) plants, the phytochemical analyses performed in this study focused on the effect of two forms of chitosan (chitosan suspension or chitosan lactate) on the total content of polyphenolic compounds and the level of some representatives of this group, i.e., flavonoids, anthocyanins, and caffeic acid derivatives.

*P. amboinicus* (some common names include Indian borage, Mexican mint, Cuban oregano, French thyme, Spanish thyme) is a perennial plant from the family Lamiaceae. The leaf raw material of this species is most often used for medicinal purposes, as it contains more than 100 substances with health-promoting properties: phenolic acids (rosmarinic acid, chlorogenic acid, caffeic acid, hydroxycinnamic acid, p-coumaric acid), flavonoids (quercetin, luteolin, apigenin, genquanine), carotenoids, steroidal glycosides, alkaloids, saponins, tannins, and phytosterols. This species also provides raw material for the extraction of essential oil, for which the chemical composition comprises germacrene, β-caryophyllene, carvacrol, thymol, camphene, zingiberene, chavicol, nerol, linalool, δ-cadinene, p-cymene, α-humulene, γ -terpinene, α-terpineol, and β-selinene. The presence of these compounds determines the wide spectrum of activity and application of fresh *P. amboinicus* leaves, as well as oils and extracts [22,23,24,25,26,27].

In folk medicine, formulations made from *P. amboinicus* leaves are used in the treatment of various ailments, e.g., cough, nasal congestion, oral diseases, colic, indigestion, hepatopathy, headaches, fever, convulsions, epilepsy, nephrolithiasis, gallstones, or rheumatism. Fresh leaves of this plant are also collected to prepare remedies for external use to soothe burns or insect and arachnid bites and to treat skin inflammation, hard-to-heal wounds, and urinary infections. Moreover, due to its high content of minerals (iron, calcium, potassium, magnesium, zinc), this plant can be consumed as a food with pro-health properties or in combination with probiotic products to restore the normal intestinal microflora [22,24,26,27,28]. The long-term use of this species in natural medicine in many countries is supported by numerous scientific studies reporting strong antibacterial, fungistatic, fungicidal, anthelmintic, diuretic, antioxidant, antimutagenic, and anticancer properties of this plant species [23,24,25,28]. Additionally, aqueous *P. amboinicus* extracts exert anti-inflammatory and analgesic effects [29], and ethanol extracts have anxiolytic and antiepileptic properties [30]. It has also been found that essential oil extracted from this species may potentially be used as a natural repellent or an agent for the control of mosquito populations [31]. However, due to the high variability of its chemical composition, further research on the biological activity and potential applications of this species is required [26].

Given the information presented above and the lack of literature reports on the potential use of chitosan for stimulation of the biosynthesis of secondary metabolites in *P. amboinicus*, the present study was carried out to compare the effectiveness of the application of a chitosan suspension and a chitosan lactate solution (differing in solubility and deacetylation degree) in the elicitation of selected phenolic compounds in the aboveground parts of this species. The hypothesis that the two forms of chitosan vary in their eliciting effects and differently influence the accumulation of phenolic compounds in *P. amboinicus* was tested. This study, for the first time, provides insight into the effect of the foliar application of chitosan on the basic physiology and chemical composition of this interesting and pharmaceutically important species.

## 2. Results

### 2.1. Content of Polyphenolic Compounds and Antioxidant Activity after Application of Chitosan

**Figure 1:**
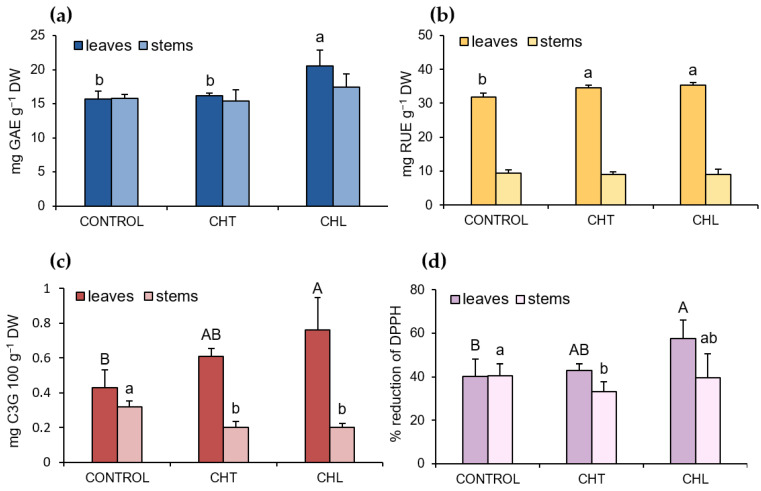
Total content of phenolics (**a**), flavonols (**b**), and anthocyanins as cyanidin 3-glycoside, C3G (**c**) and free radical scavenging activity (**d**) of *P. amboinicus* leaf and stem extracts, depending on the form of chitosan in the solution used for spraying the plants (CHT—chitosan suspension; CHL—chitosan lactate). Mean values (±standard deviation; *n* = 3) marked with different letters were statistically significantly different (*p* < 0.05). The absence of a letter designation means the absence of statistically significant differences.

The total content of polyphenolic compounds in the extracts from *P. amboinicus* leaves and stems was found to depend on the form of chitosan. Only the CHL foliar application caused a significant increase in the concentration of this group of compounds in the leaves (by 43% compared to that in the control) (Figure 1a). In comparison with the control plants, the leaves of the CHT- or CHL-treated plants showed an increase in the level of flavonols (by 8% and 11%, respectively) and anthocyanins (by 30% and 51%, respectively) (Figure 1b,c). In contrast, the level of anthocyanins in the stems declined after the application of both chitosan forms (Figure 1c). The application of chitosan did not influence the total content of phenolic compounds and flavonols in the stems (Figure 1a,b). Additionally, the extracts from the CHL-treated *P. amboinicus* leaves exhibited an increased ability to reduce DPPH radicals. However, the application of the CHT solution resulted in a decrease in the free radical-scavenging activity (FRSA) of the stems, compared to that in the control (Figure 1d).

**Figure 2:**
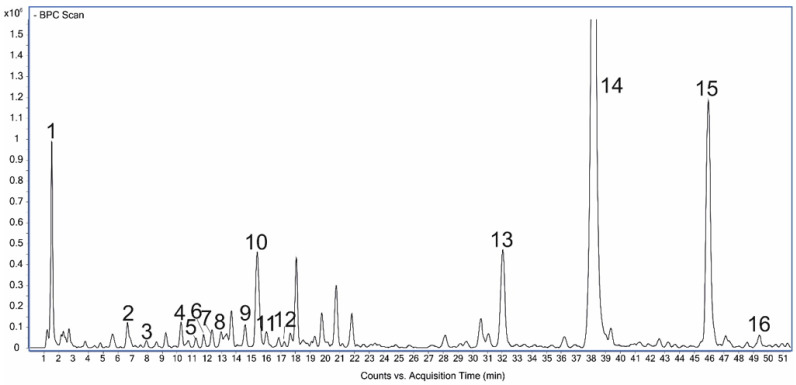
Chromatogram of *P. amboinicus* leaf extract with peaks identified via UHPLC-MS. (1) quinic acid; (2) dihydroxybenzoic acid hexoside; (3) dihydroxybenzoic acid; (4) neochlorogenic acid; (5) caffeoylglucose I; (6) caffeic acid derivative I; (7) caffeic acid derivative II; (8) caffeoylglucose II; (9) caffeoylglucose III; (10) chlorogenic acid; (11) caffeic acid; (12) caffeic acid derivative III; (13) rosmarinic acid glucoside; (14) rosmarinic acid; (15) salvianolic acid B; (16) luteolin 30-(4″-acetylglucuronide).

Given the general content of phenolic compounds and flavonols, as well as the varied response of leaf and stem tissues to chitosan application, only the *P. amboinicus* leaf extracts were subjected to further detailed analyses. All extracts had a similar qualitative profile of polyphenols, and sixteen components were identified based on mass data [m/z-H] and UV-Vis spectra obtained in the range of 200–400 nm (Table 1; Figure 2). The mass data and chromatographic parameters were compared with available standards, or the components were tentatively identified based on literature. No peaks from the new compounds were observed under the influence of CHT or CHL. The caffeic acid derivatives were predominant (Figure 2), and therefore, they were determined quantitatively. 

**Figure 3:**
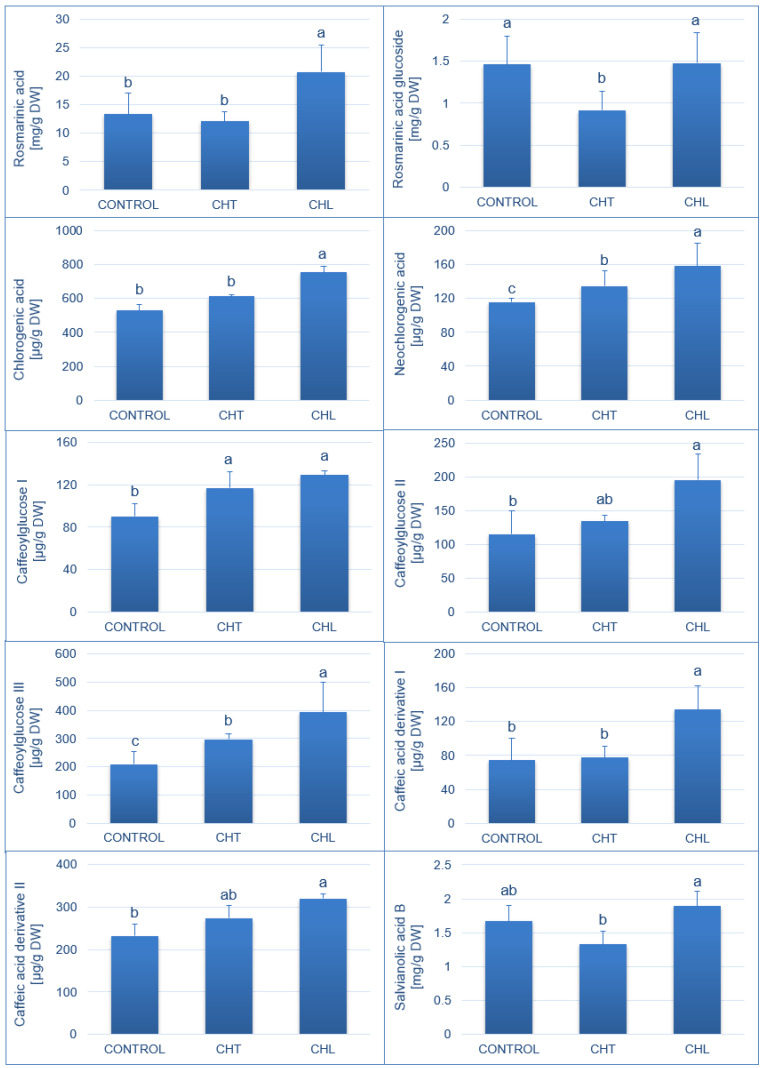
Content of selected phenolic compounds in *P. amboinicus* leaf extracts, depending on the form of chitosan used in the solutions for spraying the plants (CHT—chitosan suspension; CHL—chitosan lactate). Mean values (±standard deviation; *n* = 3) marked with different letters were statistically significantly different (*p* < 0.05).

The effect of the chitosan application on the concentration of individual caffeic acid derivatives is shown in Figure 3. Among the analysed compounds, the highest concentrations were determined in the case of rosmarinic acid, for which the level additionally increased significantly after the application of CHL (by 55% versus the control plants) but not CHT. Similarly, rosmarinic acid glucoside constituted a significant fraction of the phenolic metabolites present in the *P. amboinicus* leaves; however, its accumulation did not increase after the application of CHL but was even reduced by the CHT treatment. After the application of CHL, the level of chlorogenic and neochlorogenic acids increased by 42% and 37%, respectively, compared to that of the control. The CHT spraying treatment resulted in a 16% increase in the neochlorogenic acid level as well. The concentration of caffeoylglucose I and caffeoylglucose III increased after treatments with both forms of chitosan, whereas an increase in the caffeoylglucose II level was only observed after the application of CHL. In addition, the level of some metabolites (rosmarinic acid glucoside, salvianolic acid B) was found to decline with the CHT treatment. In general, the accumulation of most of the analysed compounds increased in plants treated with the chitosan foliar application, and CHL turned out to be a much more effective inducer of the biosynthesis and accumulation of these compounds than CHT (Figure 3).

### 2.2. Principle Component Analysis

**Figure 4:**
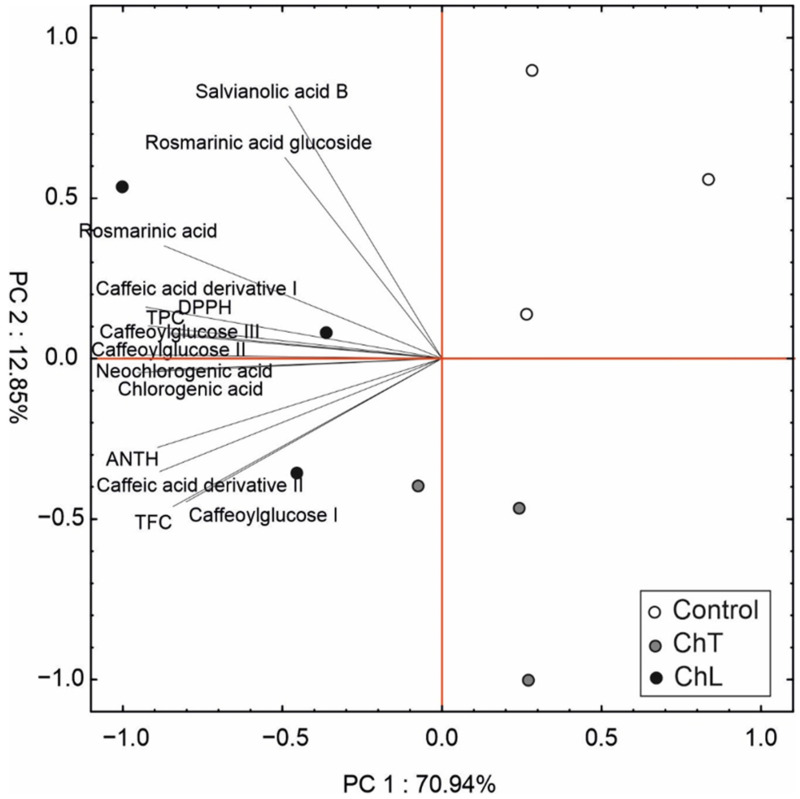
Scaled scatter plot of the principal component analysis (PCA) of selected secondary metabolites and antioxidant capacity (ANTH—anthocyanins; CHT—chitosan suspension, CHL—chitosan lactate, DPPH—antioxidant capacity, TPC—total phenolic content; TFC—total flavonol content). The length of the lines expresses the correlation between the original data and the factor axes.

The PCA of the accumulation of secondary metabolites and antioxidant capacity of the tested *P. amboinicus* leaves clearly grouped the samples into three groups according to the chitosan treatment (Figure 4). The first and second factors explained almost 84% of total variability; however, the first component defined 71% of total variability and showed negative loading by all evaluated variables. This component facilitated separation of the CHL samples from the control and partially from the CHT individuals. In turn, the second component explained approx. 14% of the total variance and was mostly positively correlated with salvianolic acid B and rosmarinic acid glucoside, but negatively correlated with TFC and caffeoylglucose I. The second component was responsible for separation of the control from the CHT individuals.

### 2.3. Dry Weight of Aboveground Parts and Selected Parameters of Chlorophyll a Fluorescence after Application of Chitosan

**Figure 5:**
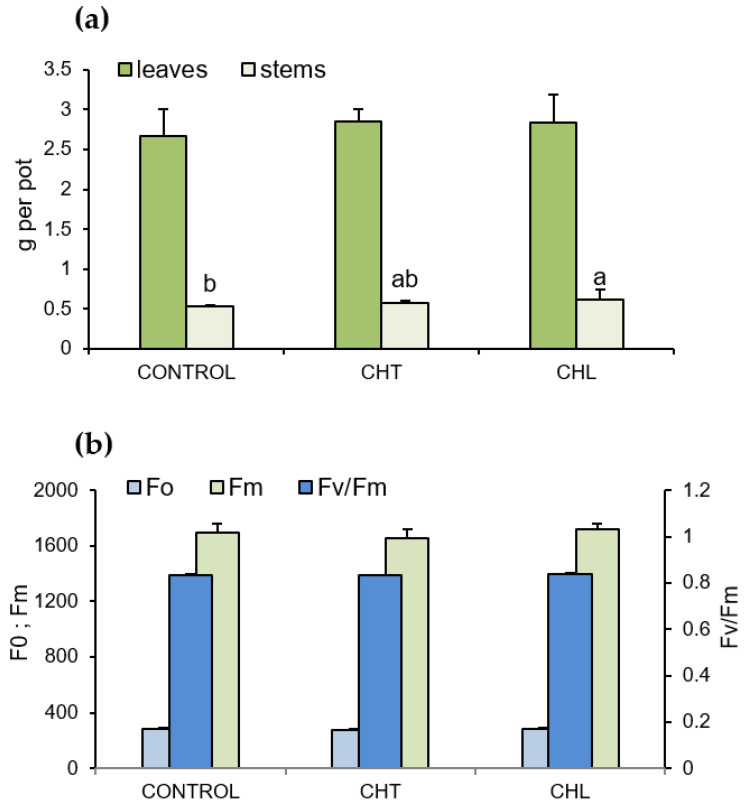
Dry matter yield of the aboveground parts (**a**) and selected chlorophyll a fluorescence indices (**b**) in P. amboinicus leaves, depending on the chitosan form in the solution used for spraying the plants (CHT—chitosan suspension; CHL—chitosan lactate). Mean values (±standard deviation; *n*_(A)_ = 3, *n*_(B)_ = 10) marked with different letters were statistically significantly different (*p* < 0.05). The absence of letter designation means the absence of statistically significant differences.

The results indicated slight differences in the effect of both forms of chitosan on the dry weight of *P. amboinicus* organs. The dry weight of the stems was higher (by 15%) compared to that of the control plants only with the CHL treatment. No such relationship was observed for the leaves (Figure 5a).

The analysis of the results of the measurement of selected chlorophyll a fluorescence indices (minimum fluorescence—F0, maximum fluorescence—Fm, and the ratio of variable to minimum fluorescence—Fv/Fm) in the *P. amboinicus* leaves showed no significant differences in the effect of chitosan on the photosynthetic apparatus functioning in the plants (Figure 5b). No visual signs of phytotoxicity of the compound were observed either.

## 3. Discussion

The progressive development of lifestyle diseases and the growing demand of pharmaceutical, food, and cosmetic industries for high-quality raw materials of plant origin have encouraged research aimed at the optimisation of plant production and analysis of bioactive compounds contained in less-common plant species. Elicitation, which involves plant defence reactions enhancing the biosynthesis and accumulation of secondary metabolites, is one of the tools used for improvements of the quality of raw materials. Chitosan is increasingly being used as a biotic elicitor in plant production. The object of the present study was *P. amboinicus*, which is highly valued in Indian, African, and Asian medicine due to its numerous pharmacological properties associated with the relatively high content of polyphenols [26]. The aim of the present study was to compare the effectiveness of the use of a chitosan suspension and chitosan lactate in the elicitation of this group of compounds in the studied species. The elicitor exhibits high biological activity, biodegradability, and non-toxicity [9], which is in line with the current environment protection trends. Moreover, the presence of the primary amino group in the chitosan molecule allows for a wide range of chemical modifications changing its solubility, charge, chelating activities, and thus biological properties [36].

In our experiment, we investigated the effect of the CHT and CHL solutions on plant growth and the function of the photosynthetic apparatus in *P. amboinicus*. The analysed parameters (biomass, chlorophyll *a* fluorescence) did not indicate a negative impact of both chitosan forms on plant growth and photosynthesis efficiency. Similarly, our previous study showed that foliar applications of CHL (100 mg/L or 500 mg/L) did not have a significant effect on the photosynthesis in *Ocimum basilicum* or slightly stimulated this process in *Melissa officinalis* [37]. Other studies not only confirmed the absence of phytotoxic effects but also indicated a beneficial effect of chitosan on photosynthesis effectiveness. As demonstrated in an experiment conducted by Xu and Mou [38], the application of moderate concentrations of chitosan (0.2% or 0.3%) to the soil improved photosynthesis parameters in lettuce. In turn, Khan et al. [39] reported an improvement in the net photosynthesis efficiency in corn and soybean leaves several days after the application of chitosan. Chitosan nanoparticles (10 ppm) were also found to increase the intensity of photosynthesis in *Coffea canephora* in greenhouse conditions [40]. 

Polyphenolic compounds are a large group of secondary metabolites with documented health-promoting properties. Given the wide spectrum of activity and the high abundance of this group of compounds in the chemical composition of *Plectranthus amboinicus* (Lour.) plants, our phytochemical analyses focused on the impact of both chitosan forms on the total polyphenol content and the level of some compounds from this group. The efficacy of elicitation was found to be largely dependent not only on the chitosan form and solubility but also on the plant organ. The foliar application of the CHL solution resulted in an increase in the total concentration of phenolic compounds (by 43% versus that in the control) in the *P. amboinicus* leaf extracts. Both chitosan forms were characterised by similar effectiveness in the induction of the accumulation of soluble flavonols in the leaves, whereas CHL increased the anthocyanin concentration more potently than CHT.

The concentration of caffeic acid derivatives in *P. amboinicus* leaves depended on the chitosan form applied. The treatment with CHL induced a significant 55%, 42%, and 37% increase in the concentration of rosmarinic, chlorogenic, and neochlorogenic acids in the leaves, respectively, relative to that in the control. In turn, the CHT foliar treatment caused a significant increase only in the concentration of neochlorogenic acid, whereas both forms of chitosan led to an increase in the level of caffeoylglucose I and caffeoylglucose III. Other studies reported the effectiveness of chitosan in the stimulation of the biosynthesis of both total polyphenols and individual compounds from this group present in herbal plants. For example, the application of 50 ppm or 200 ppm of chitosan increased the total content of phenolic compounds by 38% and 29%, respectively, in *Origanum vulgare* ssp. hirtum [41]. Similar results were obtained in experiments on *Ocimum basilicum* and *Melissa officinalis* treated with CHL solutions at concentrations of 100 mg/L or 500 mg/L [37]. The study showed that single spraying with a solution containing a lower CHL amount increased the concentration of rosmarinic acid in the analysed species most effectively. Moreover, the application of both 100 mg/L and 500 mg/L of CHL increased the total accumulation of polyphenols and anthocyanins in the lemon balm raw material. A study conducted by Fooladi Vanda et al. [42] showed the highest increase in the total content of phenolic compounds in shoot cultures of lemon balm after treatment with 100 mg/L of chitosan. However, the induction of rosmarinic acid biosynthesis did not depend on the chitosan concentration (50, 100, 150 mg/L). The effectiveness of chitosan in the elicitation of flavonoids was also demonstrated in *Isatis tinctoria* root hair cultures [43]. Similarly, studies conducted on herbal plants in greenhouse conditions indicated the effectiveness of chitosan in the stimulation of the biosynthesis of phenolic compounds and flavonoids in sage [44] and peppermint [45]. Chitosan was also reported to increase the pro-health value of fruits, e.g., apricots [46], strawberries [47], or tomatoes [48], via the stimulation of the biosynthesis of phenolic compounds. The high biological activity of chitosan and its derivatives in the stimulation of stress responses may be related to the presence of acid pectins in plant cell walls, as these compounds can bind calcium and form chain dimers. Cationic chitosan can interact with negatively charged pectin and pectin dimers modifying their supramolecular arrangement. This in turn alarms the cells of cell wall degradation, e.g., by pathogens [49], the activation of signal transduction pathways, and the initiation of a cascade of plant biochemical defence mechanisms associated with e.g., the accumulation of secondary metabolites. Moreover, chitosan can interfere positively with complex cellular networks, including cellular signalling, redox homeostasis, and transcription processes, thereby modifying plant metabolic activities [50].

Taking into account the antioxidant properties of phenolic compounds, the effect of chitosan on the FRSA of the extracts of *P. amboinicus* leaves and stems was analysed as well. The treatment with CHL, but not CHT, increased the FRSA in the leaf extracts. Similarly, chitosan was reported to increase the antioxidant activity in peppermint [45], basil [51], or sage [44] raw materials. The increase in the FRSA of extracts is most probably associated with an increase in the content of polyphenolic compounds, of which the redox properties play an important role in quenching and neutralising ROS [52]. 

One of the determinants of the success of the elicitation process is the selection of an elicitor that will be appropriate for a given plant species, as well as its concentration and exposure time [53], as the absence of phytotoxic effects is as important as the expected effectiveness. The present analyses showed that the foliar application of both chitosan forms had no negative effects on the dry weight of *P. amboinicus* organs. An exception was the CHL solution, which induced an increase in the dry weight of the stems.

The results of many studies have confirmed the effectiveness of chitosan as a plant growth-stimulating substance not only in the production of medicinal and seasoning raw materials but also in the production of fruits and vegetables. The use of this polymer was shown to have a beneficial effect on the fruit yield in e.g., haskap berries [54], strawberries [55,56], or soybeans [57]. Moreover, different concentrations of chitosan oligosaccharides were effective in stimulating the growth of *Origanum vulgare* ssp. *hirtum* in field conditions [34]. Studies carried out on okra demonstrated that the foliar application of this polymer had a positive effect on the growth and morphological features of this plant both in field conditions and in pot cultivation [58]. Similar results were obtained in experiments conducted with the use of chitosan solutions in the cultivation of other seasoning and medicinal species, i.e., *Curcuma longa* [59], *Sylibum marianum* [60], or three basil cultivars [61].

## 4. Materials and Methods

### 4.1. Experimental Design and Conditions

*Plectranthus amboinicus* (Lour.) seeds (Vilmorin Garden Company, Komorniki, Poland) were sown into 0.5 L plastic pots (20 seeds in each) filled with a potting mix, sprayed with water abundantly, and allowed to germinate. The Kronen substrate, intended for sowing seeds, transplanting seedlings, and rooting plants, was used in the experiment. The substrate contained a fine fraction of weakly and strongly decomposed high peat (pH = 6.0–6.8). The seed germination and plant growth were observed in controlled laboratory conditions, i.e., in an air-conditioned phytotron equipped with fluorescent lamps with a 14 h photoperiod, a temperature of 27 °C during the day and 23 °C at night, and 60–65% relative humidity. The surface photon flux density in the photosynthetically active range was 170–200 µmol m^−2^ s^−1^ at the level of plant tops.

On day 42 after sowing the seeds, the plants were assigned to three experimental treatment groups. Two of the groups were sprayed with aqueous solutions containing two forms of chitosan (traditional chitosan suspension—CHT or chitosan lactate—CHL) at a concentration of 200 mg/L of CHT and a dose of 10 mL per pot. The control plants received the same volume of distilled water. The spraying solutions were enriched with a 0.02% surface tension-reducing agent, Tween^®^ 20 (Sigma-Aldrich, St. Louis, MO, USA). The volume used for spraying ensured the optimal saturation of the leaves with the solutions. The chitosan forms were produced from shrimp shells; they differed substantially in their water solubility. CHL (Heppe Medical Chitosan GmbH; deacetylation degree of 80–95%) exhibited considerably higher solubility than CHT (Sigma-Aldrich; deacetylation degree of ≥75%), which formed a suspension. A varied spraying scheme was applied twice at three-day intervals. In total, 20 mL of chitosan solutions per pot was added to each experimental series. On day 10 day after the application of the first dose of the solutions, the dry matter yield of the aboveground parts was determined, the measurements of chlorophyll *a* fluorescence were carried out, and phytochemical analyses were performed.

### 4.2. Methods of Plant Material Analysis

#### 4.2.1. Preparation of Methanol Extracts

The dried plant material was ground in an electric mill. Next, 0.1 g of the raw material was mixed with 5 mL of an 80% methanol aqueous solution (*v*/*v*) and placed in an ultrasonic bath at room temperature for 30 min. The extracts were then centrifuged (5 min; 6000 rpm).

#### 4.2.2. Determination of Total Soluble Phenolic Compounds

The total content of phenolic compounds in the extracts of *P. amboinicus* leaves and stems was determined with the spectrophotometric method [62] using the Folin-Ciocalteau reagent (Chempur, Piekary Śląskie, Poland). Here, 1.9 mL of distilled water, 1 mL of F-C reagent, and 100 μL of the extract were pipetted into test tubes. The reaction mixture was shaken using an ML-962 microshaker (JWElectronics, Warsaw, Poland). After 5 min, 3 mL of a saturated Na_2_CO_3_ solution (POCH, Gliwice, Poland) was added and mixed, and the mixture was incubated at 40 °C. After 30 min, absorbance was measured spectrophotometrically at 756 nm (Cecil CE 9500, Cecil Instruments, Cambridge, UK). The total phenolic content was read from a standard curve prepared for gallic acid (Sigma-Aldrich, St. Louis, MO, USA).

#### 4.2.3. Determination of Total Soluble Flavonols

The content of flavonols in the extracts was determined with the simplified Christ-Müller method [63], which is based on the ability of flavonols to form complexes with aluminium ions. For this, 450 μL of 80% methanol, 750 μL of 2% AlCl_3_ (Acros Organics, Geel, Belgium), and 300 μL of the sample were pipetted into Eppendorf tubes. The mixture was shaken using an ML-692 microshaker (JWElectronics, Warsaw, Poland) and placed in a shaded place at room temperature for 30 min. After this time, the absorbance of the solutions was read at 425 nm (Cecil CE 9500, Cecil Instruments, Cambridge, UK). An aqueous solution of 80% methanol was the control sample (A0). The concentration of flavonols was read from the standard curve prepared for rutin (Sigma-Aldrich, St. Louis, MO, USA).

#### 4.2.4. Determination of Total Soluble Anthocyanins

The content of anthocyanins in the aboveground parts of *P. amboinicus* was determined using the spectrophotometric differential pH method [64]. Anthocyanins were extracted from 0.2 g of the plant material with 7 mL of 80% methanol (*v*/*v*) acidified to pH = 2.0 using an ultrasonic bath (25 °C, 30 min.). Next, the extracts were centrifuged (10 min; 6000 rpm). Two samples of each extract were prepared. Then, 1 mL of the analysed extract was pipetted into test tubes; next, 4 mL of buffer with pH = 1.0 was added to one of the samples, and the other sample was supplemented with 4 mL of buffer with pH = 4.5. Absorbance was read at 520 nm and 700 nm (Cecil CE 9500, Cecil Instruments, Cambridge, UK) using appropriate buffers as reagent samples. The absorbance of the analysed solutions was calculated with the following formula:

A = (A_520nm_ pH1.0 − A_700nm_ pH1.0) − (A_520nm_ pH4.5 − A_700nm_ pH4.5)

where: 

A—absorbance of the solution at a specific wavelength

The final calculations of the content of anthocyanins in the plant material, expressed in mg of cyanidin-3-glucoside (C3G) per 100 g DW, were made taking into account the molar absorption coefficient and the molecular mass of C3G, as well as the sample dilution factor. 

#### 4.2.5. Determination of Free Radical-Scavenging Activity with the DPPH Method

The free radical-scavenging activity (FRSA) of the analysed extracts was determined using the synthetic free radical DPPH (1,1-diphenyl-2-picrylhydrazyl; Sigma-Aldrich, USA). Here, 2 mL of a methanolic DPPH solution (200 μM) and 50 μL of the extract were pipetted into spectrophotometric cuvettes. Then, 15 min after the addition of the plant extract, the extinction was read at a wavelength of 517 nm (Cecil CE 9500, Cecil Instruments, UK). The DPPH solution supplemented with 50 μL of 80% methanol was the control (A0). The FRSA of the analysed extracts was calculated using the following formula [65]:

% reduction of DPPH=100 × (A0−A15)/A0

where:

A0—absorbance of the control sample

A15—absorbance 15 min after addition of the analysed sample.

#### 4.2.6. UHPLC-MS Analysis

All standards, formic acid, and MS-grade acetonitrile were obtained from Sigma-Aldrich (St. Louis, MO, USA). The extract was analysed using an ultra-high performance liquid chromatograph (UHPLC) Infnity Series II coupled with a DAD detector and an Agilent 6224 ESI/TOF mass detector (Agilent Technologies, Santa Clara, CA, USA) on an RP18 Titan column (Supelco, Sigma-Aldrich, St. Louis, MO, USA) (10 cm × 2.1 mm, 1.9 µm). The thermostat temperature was 30 °C, and the flow rate of the mobile phase was 0.2 mL/min. Water with 0.05% of formic acid (solvent A) and acetonitrile with 0.05% of formic acid (solvent B) were used as components of the mobile phase. The gradient elution program was as follows: 0–8 min from 98% A to 93% A (from 2% to 7% B), 8–15 min from 93% A to 88% A (from 7% to 12% B), 15–29 min from 88% A to 85% A (from 12% to 15% B), 29–40 min from 85% A to 80% A (from 15% B to 20% B), and 40–60 min from 80% A to 65% A (from 20% B to 35% B). Chromatograms were collected from 200 to 400 nm. The ion source operating parameters in the LC–MS analysis were as follows: drying gas temperature, 325 °C; drying gas flow, 8 L min^−1^; nebuliser pressure, 30 psi; capillary voltage, 3500 V; and skimmer, 65 V. The voltage on the fragmentator was 180 V. Ions were acquired in the range from 100 to 1300 m/z. Quantification was based on calibration curves obtained using methanol standard solutions (Sigma-Aldrich, St. Louis, MO, USA) of the identified compounds. 

#### 4.2.7. Biometric Parameters

To determine the plant dry matter, the aboveground parts were cut off from the roots at a height of several millimetres above the substrate surface. The dry matter yield was determined after drying the plant material to a constant weight at 60 °C and expressed in grams per pot.

#### 4.2.8. Measurement of Selected Parameters of Chlorophyll a Fluorescence

The parameters of chlorophyll a fluorescence were measured using a Handy-PEA portable fluorimeter (Hansatech Instruments, Pentney, UK). The following chlorophyll *a* fluorescence indices were determined: F_0_—minimum fluorescence, F_m_—maximum fluorescence, and F_v_/F_m_—ratio of variable to maximum fluorescence (F_v_ = F_m_ − F_0_), which is regarded as the most reliable and non-invasive indicator of the maximum quantum efficiency of photosystem PSII after dark adaptation [66]. Fragments of leaf blades were shaded for 15 min using special clips, and then, the measurements were carried out.

### 4.3. Statistical Analysisok

Statistical processing of numerical data provided by the laboratory analyses was carried out using Statistica ver. 13.3 (TIBCO Software Inc. 2017, Palo Alto, CA, USA). One-way analysis of variance (ANOVA) was performed, and Tukey’s post-hoc test was used to determine the significance of differences between pairs of means at the significance level α = 0.05. 

## 5. Conclusions

The comparison of the efficiency of elicitation of *P. amboinicus* with the analysed forms of chitosan showed higher bioactivity of the CHL solution than that of CHT, which was probably associated with the substantially better solubility, and thus bioavailability, of CHL and its higher deacetylation degree. The application of CHL led to a significant increase in the total content of polyphenols, soluble flavonols, anthocyanins, and most of the caffeic acid derivatives (including rosmarinic and chlorogenic acids). The changes in the level of polyphenolic compounds induced by CHT were much less pronounced; however, this chitosan form caused a significant increase in the content of caffeoylglucose I and caffeoylglucose III and a decrease in the level of rosmarinic acid glucoside. In general, despite the tendency to induce polyphenol biosynthesis by CHT, its elicitation efficiency was significantly lower than that of CHL. The CHL treatment also enhanced the FRSA of the leaf extracts, whereas the CHT treatment reduced this parameter in the stem extracts. Therefore, the foliar application of CHL solutions can be a simple, effective, inexpensive, and eco-friendly approach for optimisation of the production of *P. amboinicus* raw material in pots, yielding increased contents of certain health-promoting and antioxidant polyphenolic compounds. The present results indicate the most relevant recommendations for the practical use of chitosan as an elicitor in *P. amboinicus* cultivation and the production of plant material with increased nutraceutical value.

## Figures and Tables

**Table 1:** MS data of components identified in the *P*. *amboinicus* leaf samples in negative ionisation mode.

Peak	RT [min]	[M − H]^−^(Fragments)	Δppm	Formula	Identified	References
1	1.54	191.05648	1.92	C_7_H_12_O_6_	Quinic acid	Standard
2	6.64	315.07341 (153)	3.97	C_13_H_16_O_9_	Dihydroxybenzoic acid hexoside	[32]
3	7.89	153.01942	0.57	C_7_H_6_O_4_	Dihydroxybenzoic acid	[33]
4	10.28	353.08901 (191, 179, 135)	3.40	C_16_H_18_O_9_	Neochlorogenic acid	Standard
5	10.99	341.08831 (179, 135, 221)	1.47	C_15_H_18_O_9_	Caffeoylglucose I	[33]
6	11.32	297.06176 (179, 135, 117)	0.59	C_13_H_14_O_8_	Caffeic acid derivative I	[34]
7	12.37	297.06282 (179, 135, 117)	4.12	C_13_H_14_O_8_	Caffeic acid derivative II	[34]
8	13.00	341.08791 (179, 135, 221)	0.30	C_15_H_18_O_9_	Caffeoylglucose II	[33]
9	14.60	341.08911 (179, 135, 221)	3.81	C_15_H_18_O_9_	Caffeoylglucose III	[33]
10	15.43	353.08921 (191, 179, 135)	3.97	C_16_H_18_O_9_	Chlorogenic acid	Standard
11	16.05	179.03556	3.21	C_9_H_8_O_4_	Caffeic acid	Standard
12	17.66	297.06259 (179, 135, 117)	3.35	C_13_H_14_O_8_	Caffeic acid derivative III	[34]
13	32.49	521.13221 (359, 341, 179)	4.11	C_24_H_26_O_13_	Rosmarinic acid glucoside	[33]
14	38.52	359.07791 (179, 161)	1.86	C_18_H_16_O_8_	Rosmarinic acid	Standard [33]
15	46.41	717.14781 (519, 321, 339)	2.37	C_36_H_30_O_16_	Salvianolic acid B	[33,35]
16	49.43	503.08523 (285, 443)	4.20	C_23_H_20_O_13_	Luteolin 30-(4″-acetylglucuronide)	[33]

Caffeoylglucose and caffeic acid derivatives were quantified using a calibration curve for caffeic acid.

## Data Availability

The data presented in this study are available on request from the corresponding author.

## Sample Availability

Samples of the compounds are not available from the authors.
